# Down-regulation of EGFL8 regulates migration, invasion and apoptosis of hepatocellular carcinoma through activating Notch signaling pathway

**DOI:** 10.1186/s12885-021-08327-0

**Published:** 2021-06-15

**Authors:** Fan Wu, Fang-Yong Zhang, Guo-Qian Tan, Wei-Jia Chen, Biao Huang, Lun Yan, Hao-Lu Zhang, Shi Chen, Yang Jiao, Bai-Lin Wang

**Affiliations:** Department of Hepatobiliary Surgery, Guangzhou Red Cross Hospital, Medical College, Jinan University, Tongfu Roud 396, Guangzhou, 510220 Guangdong People’s Republic of China

**Keywords:** EGFL8, Hepatocellular carcinoma, Migration, Invasion, Apoptosis, Notch signaling pathway

## Abstract

**Background:**

Our previous studies have reported the down-regulation of EGFL8 correlates to the development and prognosis of colorectal and gastric cancer. The present study is carried out to explore the expression pattern and role of EGFL8 in hepatocellular carcinoma (HCC).

**Methods and materials:**

EGFL8 expression in 102 cases of HCC tissues matched with adjacent non-tumorous liver tissues, a normal liver cell line and three liver cancer cell lines with different metastatic capacity was detected by reverse transcription-quantitative polymerase chain reaction (RT-qPCR) and Western blot. Moreover, the clinicopathological features and prognosis of HCC patients were correlated with expression of EGFL8. Subsequently, the gain-and loss-of-function experiments were carried out to investigate the biological function of EGFL8 in HCC. We also used N-[N-(3,5-Difluorophenacetyl-L-alanyl)]-(S)- phenylglycine t-butyl ester (DAPT), an inhibitor for Notch signaling pathway, in these experiments to verify the involvement of Notch signaling pathway in the effects of EGFL8. Additionally, a mouse model was established to investigate the effect of EGFL8 on metastasis of HCC cells. The expression of Notch signaling pathway in HCC cells and xenograft mouse tumors were detected by Western blot and immunohistochemistory.

**Results:**

The expression of EGFL8 was significantly decreased in HCC tissues and cell lines and EGFL8 down-regulation correlated to multiple nodules, vein invasion, high TNM stage and poor prognosis of HCC. Interestingly, the expression levels of EGFL8 in three liver cancer cell lines were negatively associated with their metastatic capacity. In vitro and in vivo experiments indicated that EGFL8 obviously suppressed metastasis and invasion of HCC cells but slightly promoted apoptosis. Meanwhile, the expression of Notch signaling pathway was obviously suppressed in EGFL8 overexpressed HCCLM3 cells and xenograft mouse tumors generated from these cells but markedly elevated in EGFL8 depleted Hep3B cells. Furthermore, the up-regulated expression of Notch signaling pathway and effects induced by EGFL8 knockdown in Hep3B cells could be counteracted by DAPT treatment.

**Conclusion:**

The down-regulation of EGFL8 was correlated to progression and poor prognosis of HCC and regulates HCC cell migration, invasion and apoptosis through activating the Notch signaling pathway, suggesting EGFL8 as a novel therapeutic target and a potential prognostic marker for HCC.

**Supplementary Information:**

The online version contains supplementary material available at 10.1186/s12885-021-08327-0.

## Introduction

As the sixth most common cancer in globe, hepatocellular carcinoma (HCC) has become the second leading cause of male cancer death in developing countries, second only to lung cancer [1]. Despite the great improvements in treatments, the long-term survival of patients with HCC remains unsatisfactory, with a 5-year survival rate of less than 20%, mainly due to a high metastasis rate after operation [2–4]. Therefore, numerous researches have been carried out to uncover the molecular mechanisms underlying the metastasis of HCC [5]. And quite a lot of signaling pathways have been found to be involved in the metastasis of HCC including Ras/Raf/MAPK, Notch, HGF/c-Met, Wnt/β-catenin and so on [4–8]. Some other mechanism including autophagy and epigenetics modulation has also been implicated in this pathological process [9, 10]. These molecular studies have resulted in the clinical application of sorafenib, a multikinase inhibitor, however, the benefits obtained from sorafenib are very limited. Further researches are still needed to identify potential therapeutic targets for interfering the metastasis of HCC [5, 6, 8].

In a recent study, we showed the upregulation of epidermal growth factor-like domain 7 (EGFL7), a critical gene in vascular tube formation during embryogenesis [11], in HCC tissue samples and elucidated a novel role of EGFL7/FAK/ EGFR signaling pathway in metastasis of HCC, which firstly evidenced the involvement of a member of EGFL gene family in HCC [12]. As the only know paralog of EGFL7, epidermal growth factor-like domain 8 (EGFL8) shares the same overall domain structure with EGFL7 such as an EGF-like domain, a Ca^2+^ binding EGF-like domain, and a N-terminal signal peptide [11, 13], which led us to hypothesize that EGFL8 may also be involved in human cancers just like EGFL7. In fact, our subsequent studies did indicate that the expression of EGFL8 was significantly decreased in colorectal and gastric cancer tissues and this decrease correlated significantly to the progression and prognosis of these two malignancies [14, 15], suggesting EGFL8 as a novel biomarker for gastroenterological cancers. However, the expression level and biological characteristics of EGFL8 in HCC still remain unclear.

Therefore, the present study was carried out to detect the expression pattern of EGFL8 in human HCC tissues and explore the role of EGFL8 in the development of HCC in vitro as well as in vivo.

## Materials and methods

### Patients and follow-up

HCC and the corresponding adjacent non-tumorous liver tissue (ANLT) specimens were collected from 102 cases of HCC patients who underwent operation at Guangzhou Red Cross Hospital, Jinan University from February 2012 to December 2019. All tissue samples were obtained and frozen in liquid nitrogen immediately after operation and then transferred to an ultra-low temperature freezer (Meling Biology & Medical, Hefei, China) and stored at − 80 °C until detection. The diagnoses of HCC were confirmed by histopathological observation. The clinicopathologic data of the patients including age, gender, etiologies, liver cirrhosis status, serum α-fetoprotein (AFP) level, maximal tumor size, tumor cell differentiation (Edmondson- Steiner grade), tumor nodule number, capsular formation, vein invasion (including portal vein invasion, venous invasion or microscopic vessel invasion), and tumor node metastasis (TNM) stage were also collected. Follow-up data were obtained after hepatic resection for all 102 patients. The follow-up period was defined as the interval between the date of operation and that of patient’s death or the last follow-up. Deaths from other causes were treated as censored cases. Recurrence and metastasis were diagnosed by clinical examination, serial AFP level mensuration, and ultrasonography or computed tomography (CT) scan. Prior written informed consent of all patients involved in the study was obtained before operation and the protocol of the present study was approved by the Ethics Committee of Guangzhou Red Cross Hospital.

### Cell culture and reagents

HL-7702 normal liver cell line and HCCLM3, Hep3B, HepG2 liver cancer cell lines were purchased from GeneChem Technology (Shanghai, China) and all cultured at 37 °C in a humidified incubator (SANYO, Osaka, Japan) with 5% CO_2_ by using high glucose DMEM (Dulbecco’s modified Eagle’s medium) containing 10% fetal bovine serum (FBS, Invitrogen, Frederick, MD, USA). The rabbit polyclonal antibody against human EGFL8, Notch1, NICD, Hes1 and Hey1 were purchased from Abcam (Cambridge, UK). The mouse monoclonal antibody against human GAPDH was purchased from Santa Cruz Biotechnology (Santa Cruz, CA, USA). The γ-secretase inhibitor N-[N-(3,5-Difluorophenacetyl- L-alanyl)]-(S)-phenylglycine t-butyl ester (DAPT, CAS: 208255–80-5) was also purchased from Abcam and the dimethylsulfoxide (DMSO) was purchased from Sigma (St. Louis, MO, USA).

### RNA preparation and reverse transcription-quantitative polymerase chain reaction (RT-qPCR)

The total RNA of HCC tissues, adjacent non-tumorous liver tissues (ANLTs), liver cancer cell lines was extracted by using TRIzol reagent (Invitrogen, Carlsbad, CA, USA) as previously described [14–16]. The protocol of the first-strand cDNA generation and was also performed as described previously [16]. The quantitative polymerase chain reaction (qPCR) was consisted of 40 cycles after an initial denaturation step (95 °C for 3 min) and every cycle including 95 °C for 5 s and then 60 °C for 30 s. As an internal control, β-actin gene in the same samples was also detected. The primers for EGFL8 and β-actin qPCR amplification were designed by Primer Premier 5.0 software (Premier Biosoft International, Palo Alto, CA, USA). EGFL8: forward, 5′-CCCGCTCCACTACAACGAGT-3′; reverse, 5′-AACGCGGTACATGGTCCTGT- 3′. Beta-actin: forward, 5′-GCATGGGTCAGAAGGATTCCT-3′; reverse, 5′-TCG- TCCCAGTTGGTGACGAT-3′. All the qPCR analyses were performed in triplicate and the results were calculated by 2^-ΔΔCt^ method. The relative expression of EGFL8 mRNA in tissue samples and liver cancer cell lines were normalized to β-actin and the EGFL8 expression was defined as down-regulation when the relative expression of EGFL8 in HCC tissue < the relative expression of EGFL8 in the corresponding ANLT tissue. Egfl8 expression in HCC specimens were also divided into high EGFL8 expression group (EGFL8 expression level ≥ median of EGFL8 expression levels in all HCC specimens) and low EGFL8 expression group (EGFL8 expression level < median of EGFL8 expression levels in all HCC specimens).

### Western blot

Total protein of HCC tissues, ANLTs and liver cancer cell lines was extracted and separated by SDS-PAGE and subsequently transferred onto PVDF (Polyvinylidene Fluoride) membrane (Merck Millipore, Etobicoke, Ontario, Canada), which were incubated successively with primary antibody against EGFL8, Notch1, NICD, Hes1, Hey1 and the corresponding secondary antibody. GAPDH protein in the same samples was also detected as a loading control. The expression levels of EGFL8, Notch1, NICD, Hes1 and Hey1 proteins were normalized to GAPDH. Down-regulation of EGFL8 protein was defined as positive when the relative expression of EGFL8 protein in HCC tissue < the relative expression of EGFL8 protein in the corresponding ANLT tissue.

### Lentivirus-mediated EGFL8 transfection

Full-length human EGFL8 cDNA was amplificated by polymerase chain reaction as described previously [17]. Then EGFL8 expression vector was generated by subcloning EGFL8 cDNA into the GV143 plasmid (purchased from GeneChem). The package and infection of lentivirus containing EGFL8 expression vector or empty vector were also described previously [17]. The HCCLM3 cells infected with lentivirus containing EGFL8 expression vector were named as HCCLM3^EGFL8^ and those HCCLM3 cells infected with lentivirus containing empty vector were named as HCCLM3^Vector^. And Western blot analysis was subsequently used to determine the expression levels of EGFL8 protein in HCCLM3^EGFL8^ and HCCLM3^Vector^ cells.

### Lentivirus-mediated short hairpin RNA (shRNA)

The GV248 shRNA expressing vector was purchased from GeneChem Technology (Shanghai, China). The sequences of shRNA targeting EGFL8 (selected from three putative candidate sequences, data not shown) were: sense: 5′-CCGGCAACCAGTGCCAGCATACTCACTCGAGTGAGTATGCT- GGCACTGGTTGTTTTTG-3′; anti- sense: 5′-AATTCAAAAAGAGTTGGTA- CTGGATCACATTCTCGAGAATGTGATCCAGTACCAACTC-3′. And the negative control shRNA sequences (not targeting any known gene) were: sense: 5′-CCGGTTCTCCGAACGTGTCACGTTTCAAGAGAACGTGACAC- GTTCGGAGAATTTTTG-3′; antisense: 5′-AATTCAAAAATTCTCCGAACGT- GTCACGTAAGTTCTCTACGTGACACGTTCGGAGAA-3′. The package and infection of lentivirus containing shRNA sequence targeting EGFL8 or negative control sequences were described previously [18]. The Hep3B cells infected with lentivirus containing shRNA sequence targeting EGFL8 were named as Hep3B^shEGFL8^ and those Hep3B cells infected with lentivirus containing negative control sequence were named as Hep3B^shCtrl^. The expression levels of EGFL8 protein in Hep3B^shEGFL8^ and Hep3B^shCtrl^ cells were also detected by Western blot analysis.

### Wound healing assay

Liver cancer cells were seeded on 96-well plates (3 × 10^4^ cells/well). The cells were incubated for 24 ~ 48 h and when the confluency reached about 80%, a linear wound was made by a cell scraper (1.2 mm width) across the cell monolayer. Then the cells were washed twice and incubated with the high glucose DMEM without FBS. And photographs were taken at 0, 24 and 72 h by a microscope (MicroPublisher 3.3RTV, OLYMPUS, Tokyo, Japan). The 24 h / 72 h wound closure rate = (0 h width of wound - 24 h / 72 h width of wound) / 0 h width of wound × 100%. The scattered spherical cells in the scratch wound of the original image, which were scratched out or washed out by PDS and adhered again in the scratch wound after PDS washing, did not influence the assessment of the results. Experiments were carried out in triplicate.

### Transwell invasion assay

Transwell invasion assay were carried out according to the same methods described previously [19]. These experiments were performed in triplicate.

### MTT assay and cell apoptosis analysis

The protocol of MTT assay and cell flow cytometric for apoptosis analysis were also described previously [17, 18, 20].

### HCC metastatic mouse model

A total of 20 nude mice, 6 to 8 weeks old, were purchased from Tongji University Experimental Animal Center (Shanghai, China) and reared in cages under SPF (specific pathogen free) conditions at 21–25 °C with 40–70% humidity, 12/12 light cycles and free access to food and water. These mice were randomized into the HCCLM3^EGFL8^ group and the HCCLM3^Vector^ group (10 mice/group). HCCLM3^EGFL8^ or HCCLM3^Vector^ cells were subcutaneously inoculated into the mice in HCCLM3^EGFL8^ or HCCLM3^Vector^ group on their right upper flank regions (1 × 10^7^ cells/mouse). Since the subcutaneous tumors emerged from these mice, the volume (*V*) of tumors was measured every other day until the ninth measurement (in 16 days after the emergence of the tumor) and calculated by using the formula: *V* = *a* × *b*^2^/2, in which *a* means the largest tumor diameter and *b* means the smallest one. During the whole time of experiment, the health and behavior of these mice were observed every other day. If the tumor burden of a mouse was evaluated to be high or a mouse was found to be difficult to feed, this mouse would be euthanized early. However, no early euthanasia was actually executed. After the ninth measurement of the subcutaneous tumors, which was in 26 ~ 31 days after HCCLM3^EGFL8^ or HCCLM3^Vector^ cells inoculation, all 20 mice were euthanized with pentobarbital sodium by intraperitoneal injection at a dose of 150 mg/kg and then the euthanasia was confirmed by cervical dislocation. Subsequently, the subcutaneous tumors were dissected out and cut into 4 μm thick slices for hematoxylin and eosin (H&E) staining and immunohistochemistry. Lung of each mouse was made into serially sections and observed under a microscope after H&E stain as described previously [12]. Once metastatic HCC cells were found on any slide of lung sections of a mouse, which was considered as lung metastasis positive. All animal experiments were designed to minimize pain or discomfort to the animals and complied with the ARRIVE guidelines and the AVMA guidelines for the euthanasia of animals (2013 Edition). The animals were acclimatized to laboratory conditions (23 °C, 12 h/12 hlight/dark, 50% humidity, ad libitum access to food and water) for 2 wk. prior to experimentation. All the experiment protocols involving animals were reviewed and approved by the Ethics Committee of Guangzhou Red Cross Hospital and this study was carried out in compliance with the ARRIVE guidelines.

### Immunohistochemistry assay

As described previously, the expression of EGFL8 and Notch1 in mouse xenograft tumors was determined by immunohistochemistry (IHC) assay [12, 17, 18]. Briefly, the sections made from xenograft tumors were deparaffinized, rehydrated and incubated with 3% H_2_O_2_. Then, these sections were put in citrate buffer (0.01 M, pH 6.0) and heated by a microwave oven at high power for two times, each for 7 min, for antigen retrieval. After rinsing with PBS, the sections were incubated successively with primary antibodies and HRP-conjugated second antibodies. Finally, the sections were visualized by using 3,3-diaminobenzidine tetrahydrochloride (DAB) and counterstained with hematoxylin.

### DAPT treatment

To verify the role of Notch signaling pathway in the biological effects of EGFL8 on liver cancer cells, EGFL8 depleted Hep3B cells were treated with DAPT (50 μM, dissolved by DMSO), an inhibitor of Notch signaling pathway, for 48 h [21].

### Statistics analyses

The expression levels of EGFL8 in HCC tissues were compared with those in ANLTs by Mann-Whitney test. Data come from three or five independent experiments are presented as mean ± SEM (standard error of mean). The differences between two groups were examined by unpaired *t* test. The comparisons of multi-group were performed by one-way analysis of variance (ANOVA) with Tukey test as post hoc test. The growth curves of liver cancer cells and xenograft tumors were compared by ANOVA with Tukey test. Survival curves were constructed using the Kaplan-Meier method and evaluated using the Log-Rank test. These analyses were all completed by using Graphpad Prism 7.0 software (Graphpad Software, La Jolla, CA, U.S.A.). All analyses were two-sided and *P* < 0.05 was considered as significant.

## Results

### The decreased expression of EGFL8 in HCC tissues and its association with the clinicopathological features and survival of HCC

**Fig. 1:**
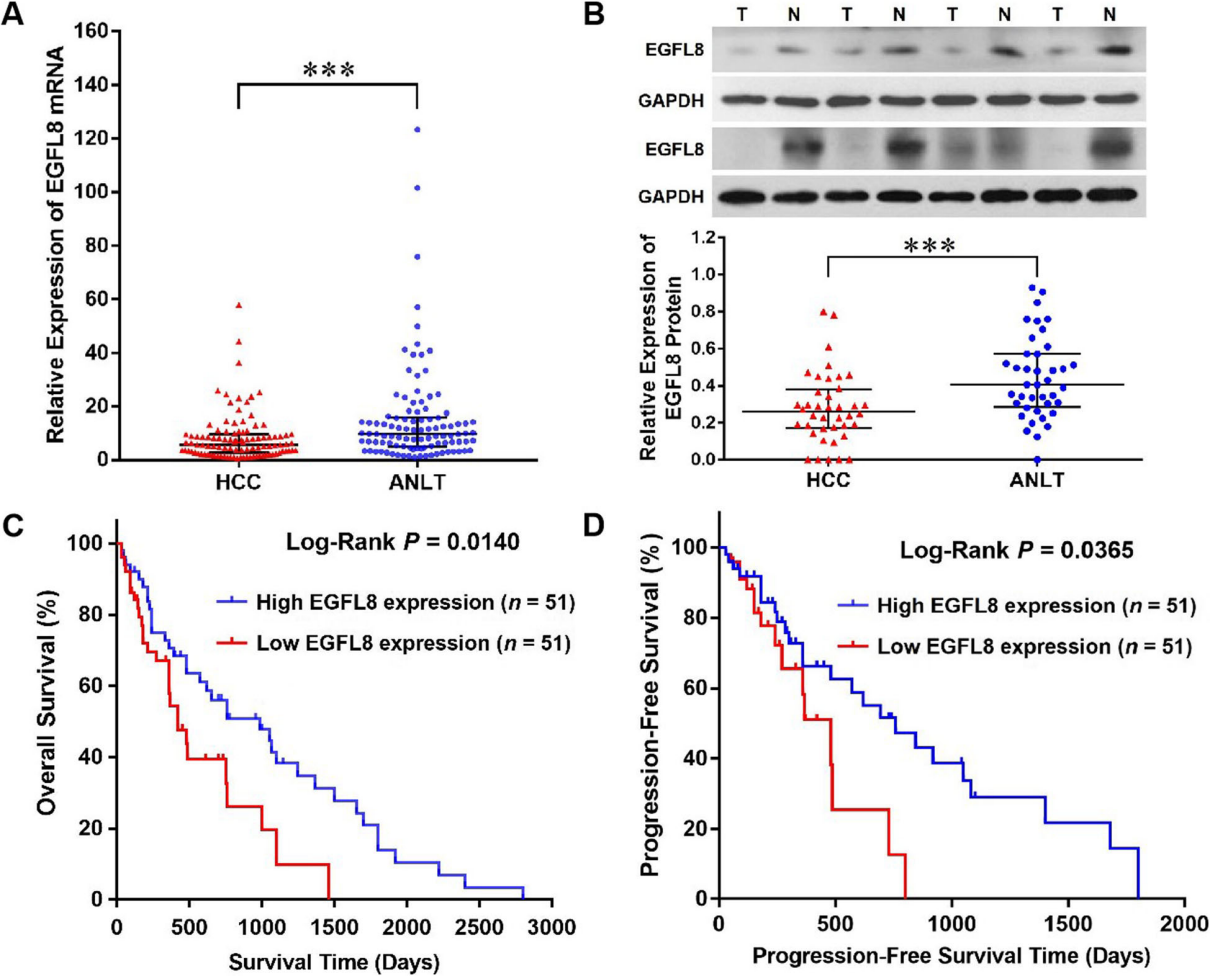
The expression of EGFL8 in HCC tissues and its association with prognosis of HCC patients. **a** The reverse transcription-quantitative polymerase chain reaction (RT-qPCR) results obtained from 102 cases of HCC tissues and matched ANLTs showed that EGFL8 mRNA expression was down-regulated in HCC tissues (the long bar in this figure represents median and the short bars represent 25 and 75% percentiles of the expressions of EGFL8). ***, *P* < 0.001. **b** The representative Western blot results showed that Egfl8 protein in HCC tissues was significantly lower than those in ANLTs (the long bar in this figure represents median and the short bars represent 25 and 75% percentiles of the expressions of EGFL8). T, HCC tissues; N, ANLTs; ***, *P* < 0.001. **c** Estimated overall survival according to the expression of EGFL8 mRNA in 102 cases of HCC tissues (the Kaplan-Meier method). Log-Rank test shows that HCC patients within low EGFL8 expression group had poorer overall survival than those in high EGFL8 expression group (*P* = 0.0140). **d** Progression free survival was analyzed in the same cohort of HCC patients and the results showed that HCC patients in low EGFL8 expression group also had poorer progression free survival than those in high EGFL8 expression group (*P* = 0.0465)

The expression level of EGFL8 in HCC tissues was significantly decreased compared with the corresponding ANLT specimens (median, 5.651 versus 9.475; *P* < 0.0001) and the expression of EGFL8 was down-regulated in 73.81% (76/102) of HCC patients (Fig. 1a). To verify the results of RT-qPCR, we also detected the expression of EGFL8 protein in 40 cases of HCC and the corresponding ANLTs, which is included in 102 cases of HCC and ANLTs, by Western blot and the results evidenced the decreased expression of EGFL8 protein in HCC tissues (median, 0.260 versus 0.406, *P* < 0.001; Fig. 1b) and down-regulation of EGFL8 protein was positive in 70% (28/40) of HCC patients. Our results further showed that EGFL8 down- regulation correlated significantly to multiple tumor nodes (*P* = 0.019), vein invasion (*P* = 0.012), and high TNM stage (*P* = 0.031) of HCC (Table 1). However, there was no significant correlation between EGFL8 down-regulation and the other clinicopathologic features of HCC. To explore the prognostic implication of EGFL8 expression in HCC, we divided all 102 cases of HCC patients into low EGFL8 expression group (*n* = 51) and high EGFL8 expression group (*n* = 51) according to the results of RT-qPCR and compared the overall and progression-free survival between these two groups. Our results showed that the HCC patients within low EGFL8 expression group had either worse overall survival (median survival time, 420 days versus 985 days, *P* = 0.0140; Fig. 1c) or worse progression free survival (median progression free survival time, 380 days versus 760 days, *P* = 0.0365; Fig. 1d) than those within high EGFL8 expression group.

**Table 1:** Correlations between the down-regulation of EGFL8 and clinico- pathological features of hepatocellular carcinoma

			Down-regulation of Egfl8		
Clinicopathological features	Variables	*n*	Positive	Negative	*P*-Value^†^
Gender	Male	80	60	20	0.828
	Female	22	16	6	
Age	≥ 65 years	44	34	10	0.650
	< 65 years	58	42	16	
Etiology	HBV infection	71	54	17	0.154
	HCV infection	9	6	3	
	Other or rare cause	22	12	10	
Liver cirrhosis	Presence	65	51	14	0.245
	Absence	37	25	12	
Serum AFP level	≥ 20 μg/L	60	43	17	0.494
	< 20 μg/L	42	33	9	
Maximal tumor size	≥ 5 cm	63	50	13	0.168
	< 5 cm	39	26	13	
Edmondson-Steiner grade	I – II	71	54	17	0.626
	III – IV	31	22	9	
Capsular formation	Presence	57	39	18	0.169
	Absence	45	37	8	
Tumor nodule number	Solitary	61	40	21	0.019^*^
	Multiple (≥2)	41	36	5	
Vein invasion	Presence	46	40	6	0.012^*^
	Absence	56	36	20	
TNM stage^a^	I – II	52	34	18	0.031^*^
	III – IV	50	42	8	

^†^ The *P*-values were obtained from Chi-square test or Fisher’s exact test and all tests were two-sided. ^a^TNM classification is according to 8th Edition of the AJCC (American Joint Committee on Cancer) Cancer Staging Manual. *, *P* < 0.05. EGFL8:epidermal growth factor-like domain; *AFP* α-fetoprotein, *HBV* Hepatitis B virus, *HCV* Hepatitis C virus

### The expression of EGFL8 and its overexpression and knockdown in liver cancer cell lines

**Fig. 2:**
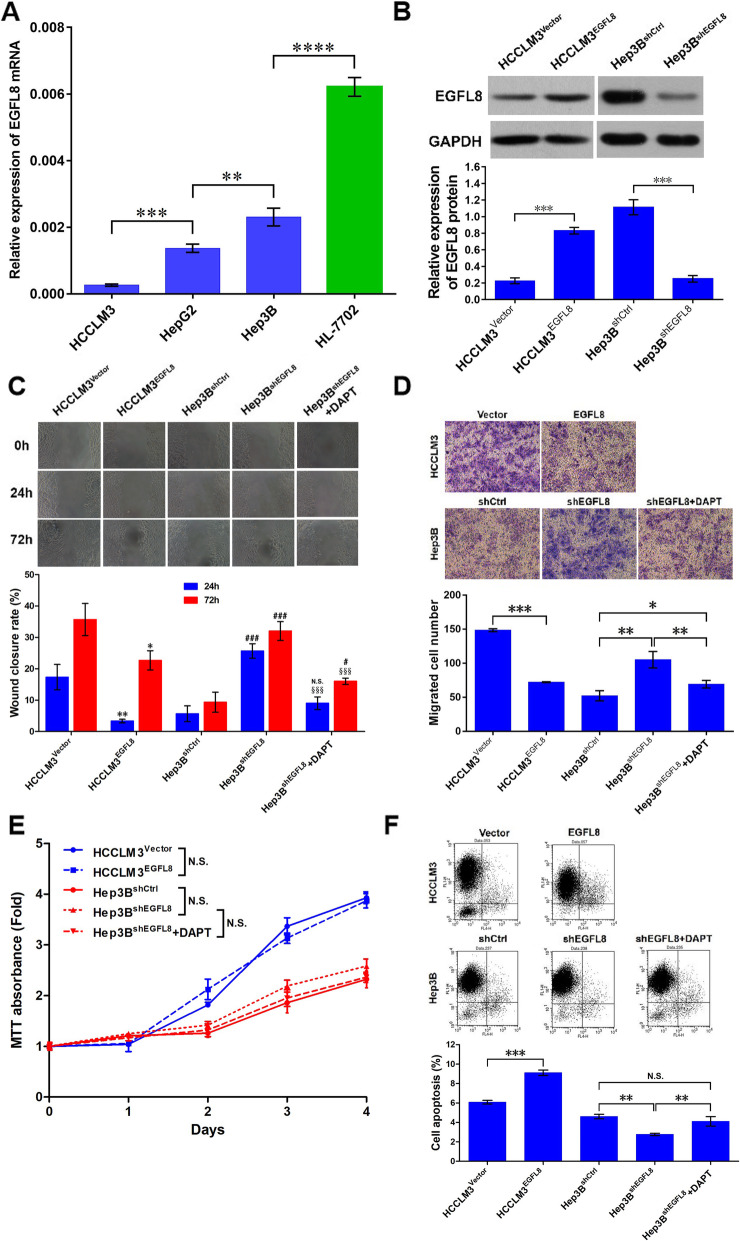
The gain- and loss-of-function experiments indicated the involvement of EGFL8 in migration, invasion and apoptosis of HCC cells. **a** The expression of EGFL8 in HCCLM3, Hep2B and Hep3B liver cancer cell lines was significantly lower than that in HL-7702 normal liver cell line. EGFL8 expression level was lower in high-metastatic liver cancer cell line HCCLM3 than low-metastatic liver cancer cell lines HepG2. EGFL8 expression in the latter was also lower than almost non-metastatic liver cancer cell line Hep3B. Data were showed as mean ± SEM. **, *P* < 0.01; ***, *P* < 0.001; ****, *P* < 0.0001. **b** The overexpression or inhibition efficiency of EGFL8 protein in HCCLM3 or Hep3B cell lines after lentivirus infection was determined by Western blot. GAPDH was also measured as loading control. Data were showed as mean ± SEM. ***, *P* < 0.001. Full-length blots are presented in Supplementary Fig. 1. **c** Wound healing assay showed that 24 h and 72 h wound closure rates were all remarkably reduced in HCCLM3^EGFL8^ cells but notedly elevated in Hep3B^shEGFL8^ cells. DAPT (N-[N-(3,5-Difluorophenacetyl-L- alanyl)]-(S)-phenylglycine t-butyl ester) treatment could significantly decrease wound closure rate of Hep3B^shEGFL8^ cells, whatever 24 h or 72 h. Magnification, × 200. Data were showed as mean ± SEM. *, *P* < 0.05, **, *P* < 0.01, versus HCCLM3^Vector^ cells; ^#^, *P* < 0.05, versus Hep3B^shCtrl^ cells; ^###^, *P* < 0.001, versus Hep3B^shCtrl^ cells; N.S., no significance, versus Hep3B^shCtrl^ cells; ^§§§^, *P* < 0.001, versus Hep3B^shEGFL8^ cells. **d** The results of Transwell invasion assay showed the number of HCCLM3^EGFL8^ cells invaded through the matrigel was significant decreased than that of HCCLM3^Vecter^ cells. However, the number of Hep3B^shEGFL8^ cells permeated through the matrigel was obviously increased than that of Hep3B^shCtrl^ cells and DAPT treatment could significantly reduce this number. Magnification, × 200. Data were showed as mean ± SEM. **, *P* < 0.01; ***, *P* < 0.001. **e** The MTT assay showed there was no significant difference between the growth curves of HCCLM3^EGFL8^ and HCCLM3^Vector^ cells. Although a very subtle increase of proliferation could be found in Hep3B^shEGFL8^ cells compared with Hep3B^shCtrl^ cells, which did not lead to a significant difference between the proliferation of these two groups of cells. DAPT treatment did not exhibit a significant influence on the proliferation of Hep3B^shEGFL8^ cells. N.S., no significance. **f** The apoptosis levels of HCC cells were determined by flow cytometric analysis and the cell percentage in each quadrant was shown. The percentage of apoptotic cells in every group of HCC cell was shown by histogram. Data were showed as mean ± SEM. **, *P* < 0.01; ***, *P* < 0.001. N.S., no significance

RT-qPCR results showed that the expression levels of EGFL8 in three liver cancer cell lines were also significantly lower than that in HL-7702 liver cell line. Interestingly, EGFL8 expression level in high-metastatic liver cancer cell line HCCLM3 was obviously lower than low-metastatic liver cancer cell lines HepG2, in which the expression level of EGFL8 was also lower than almost non-metastatic liver cancer cell lines Hep3B, suggesting the involvement of EGFL8 in metastasis of liver cancer cells (Fig. 2a). Therefore, EGFL8 was overexpressed by lentivirus-mediated gene transduction in HCCLM3 cells and depleted by lentivirus-mediated short hairpin RNA (shRNA) in Hep3B cells. Western blot results subsequently indicated that EGFL8 protein levels in HCCLM3^EGFL8^ and Hep3B^shEGFL8^ cells were increased by 3.66 times (0.2267 ± 0.0203 versus 0.8300 ± 0.0231, *P* < 0.0001) and decreased by 4.45 times (1.113 ± 0.0524 versus 0.2500 ± 0.0231, *P* = 0.0001) compared with the corresponding negative control cells, respectively (Fig. 2b).

### EGFL8 suppresses the migration and invasion of HCC cells

Since the expression of EGFL8 correlated with the metastatic potential of liver cancer cells (Fig. 2a), we firstly investigated the effects of EGFL8 on HCC cell migration and invasion. As shown in Fig. 2c, though a few scattered spherical cells were found in the scratch wound, the wound healing assay showed that the closure rate of wound in HCCLM3^EGFL8^ was significantly lower than HCCLM3^Vector^ (for 24 h: 3.33% ± 0.33% versus 17.33% ± 2.33%, *P* = 0.004; for 72 h: 22.67% ± 1.76% versus 35.67% ± 2.96%, *P* = 0.0026) but higher in Hep3B^shEGFL8^ cells than Hep3B^shCtrl^ cells (for 24 h: 25.67% ± 1.33% versus 5.67% ± 1.45%, *P <* 0.001; for 72 h: 38.67% ± 2.03% versus 9.33% ± 1.86%, *P <* 0.001), indicating a suppressing role of EGFL8 in migration of HCC cells. Subsequently, we performed a Transwell invasion assay and the results showed that the number of HCCLM3^EGFL8^ cells invaded passed through the matrigel was much less than that of HCCLM3^Vector^ cells (72.33 ± 3.33 versus 148.70 ± 7.21, *P* < 0.001; Fig. 2d) but the number of Hep3B^shEGFL8^ cells invaded passed through the matrigel was more than Hep3B^shCtrl^ cells (105.30 ± 6.98 versus 52.33 ± 4.26, *P* = 0.0008; Fig. 2d), further suggesting a suppressing role of EGFL8 on the metastasis and invasion of HCC cells.

### EGFL8 does not affect the proliferation of HCC cells but slightly promotes apoptosis

The MTT results showed no obviously difference between the growth curves of HCCLM3^EGFL8^ and HCCLM3^Vector^ cells (Fig. 2e). Similarly, there was also no significant difference between the growth curves of Hep3B^shEGFL8^ and Hep3B^shCtrl^ cells, although the proliferation rates of Hep3B^shEGFL8^ cells seem slightly higher than Hep3B^shCtrl^ cells in every timepoint (Fig. 2e). On the other hand, the apoptosis level increased in HCCLM3^EGFL8^ cells than HCCLM3^Vector^ cells (6.08% ± 0.11% versus 9.11% ± 0.16%, *P* < 0.001) and decreased in Hep3B^shEGFL8^ cells than Hep3B^shCtrl^ cells (2.77% ± 0.07% versus 4.60% ± 0.13%, *P* = 0.001; Fig. 2f), implicating a slightly promotive effect of EGFL8 on apoptosis of liver cancer cells.

### EGFL8 overexpression suppresses the metastasis of HCC cells in vivo

**Fig. 3:**
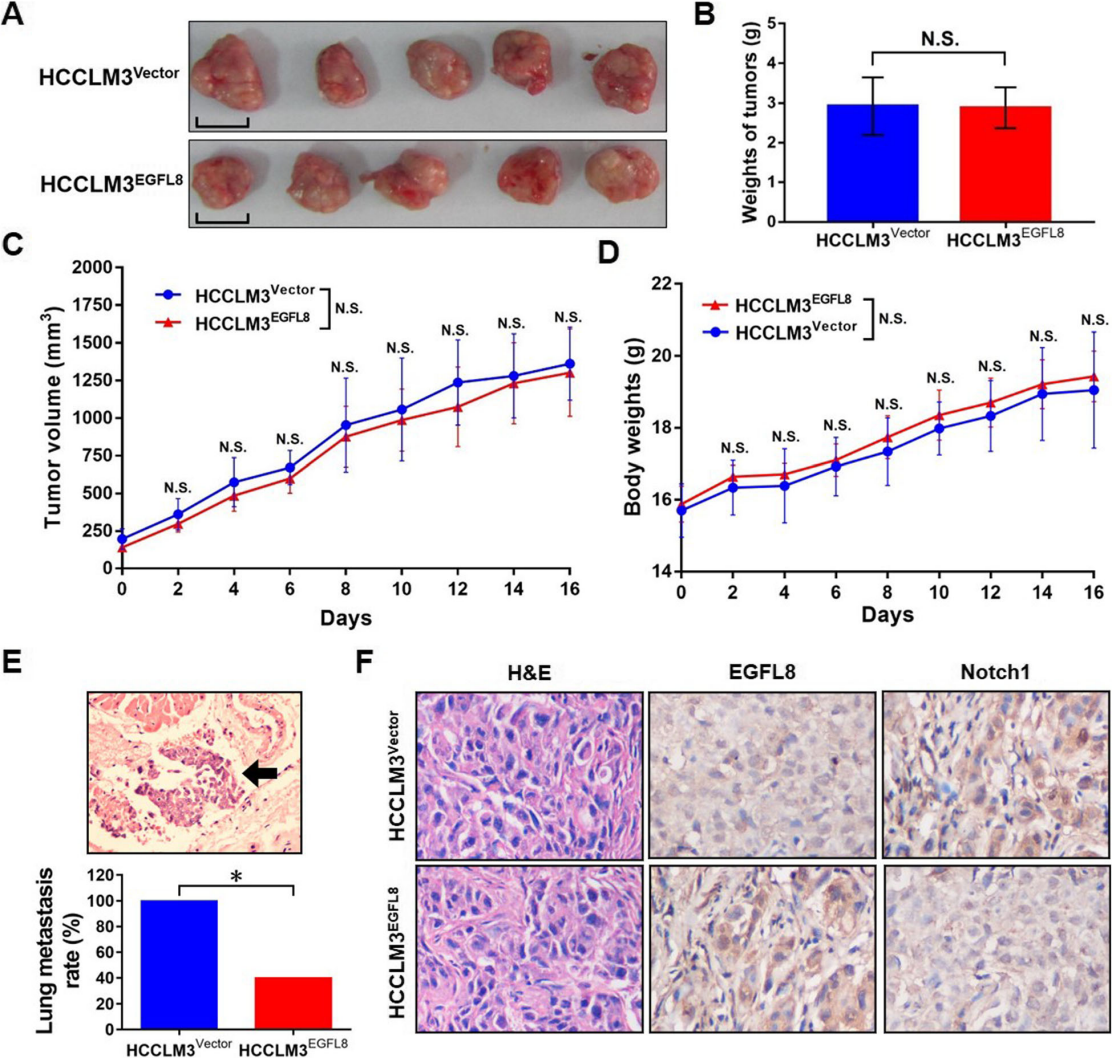
EGFL8 overexpression inhibits the metastasis of HCCLM3 cells in vivo. **a** The presentative images for the mouse xenograft tumors came from HCCLM3^Vecter^ and HCCLM3^EGFL8^ cells respectively. The scales in the images represent 1 cm. **b** No significant difference was found in the weights of xenograft tumors between HCCLM3^Vecter^ and HCCLM3^EGFL8^ groups. N.S., no significance. **c** There was no obvious difference in the growth curves of the xenograft tumors between HCCLM3^Vecter^ and HCCLM3^EGFL8^ groups. “Days” under the X axis means the time after the emergence of the subcutaneous tumors. N.S., no significance. **d** No difference was found in the body weights of the mice between HCCLM3^Vecter^ and HCCLM3^EGFL8^ groups. “Days” under the X axis means the time after the emergence of the subcutaneous tumors. N.S., no significance. **e** The lung metastasis rate of HCCLM3^EGFL8^ cells was obviously lower than that of HCCLM3^Vecter^ cells. Black arrow in the image indicates the metastatic HCCLM3 cell mass in lung tissues of the mice. Magnification, × 400. N.S., no significance; *, *P* < 0.05. **f** The representative results of H&E staining and immunohistochemistry assay for the expression of EGFL8 and Notch1 in xenograft mouse tumors generated from HCCLM3^Vecter^ and HCCLM3^EGFL8^ cells. Magnification, × 400

To confirm the results obtained from in vitro studies, we explored the in vivo relevance of EGFL8 in HCC by a mouse HCC metastasis model. The results showed no significant difference in the sizes, weights or growth curves of xenograft tumors between HCCLM3^EGFL8^ cells and HCCLM3^Vector^ cells groups (Fig. 3a-c). Meanwhile, we also have not found any significant difference in body weights of mice between HCCLM3^EGFL8^ and HCCLM3^Vector^ groups (Fig. 3d). Since the in vitro data suggesting a role of EGFL8 in metastasis of liver cancer cells, we further detected the lung metastasis of HCCLM3 cells in these mice. The lung metastasis of HCCLM3 cells was found in four mice in the HCCLM3^EGFL8^ group (four of ten, 40%), much less than the lung metastasis rate in the HCCLM3^Vector^ group (ten of ten, 100%; *P* = 0.011), as shown in Fig. 3e. Additionally, H&E stain confirmed the diagnosis of HCC for the subcutaneous xenograft tumors of two groups of mice and the results of IHC assay showed a significantly increased EGFL8 expression and an obviously decreased Notch1 expression in the tumors of HCCLM3^EGFL8^ cells compared with those tumors of HCCLM3^Vector^ cells (Fig. 3f).

### EGFL8 regulates metastasis, invasion and apoptosis of HCC cells through suppressing the notch signaling pathway

**Fig. 4:**
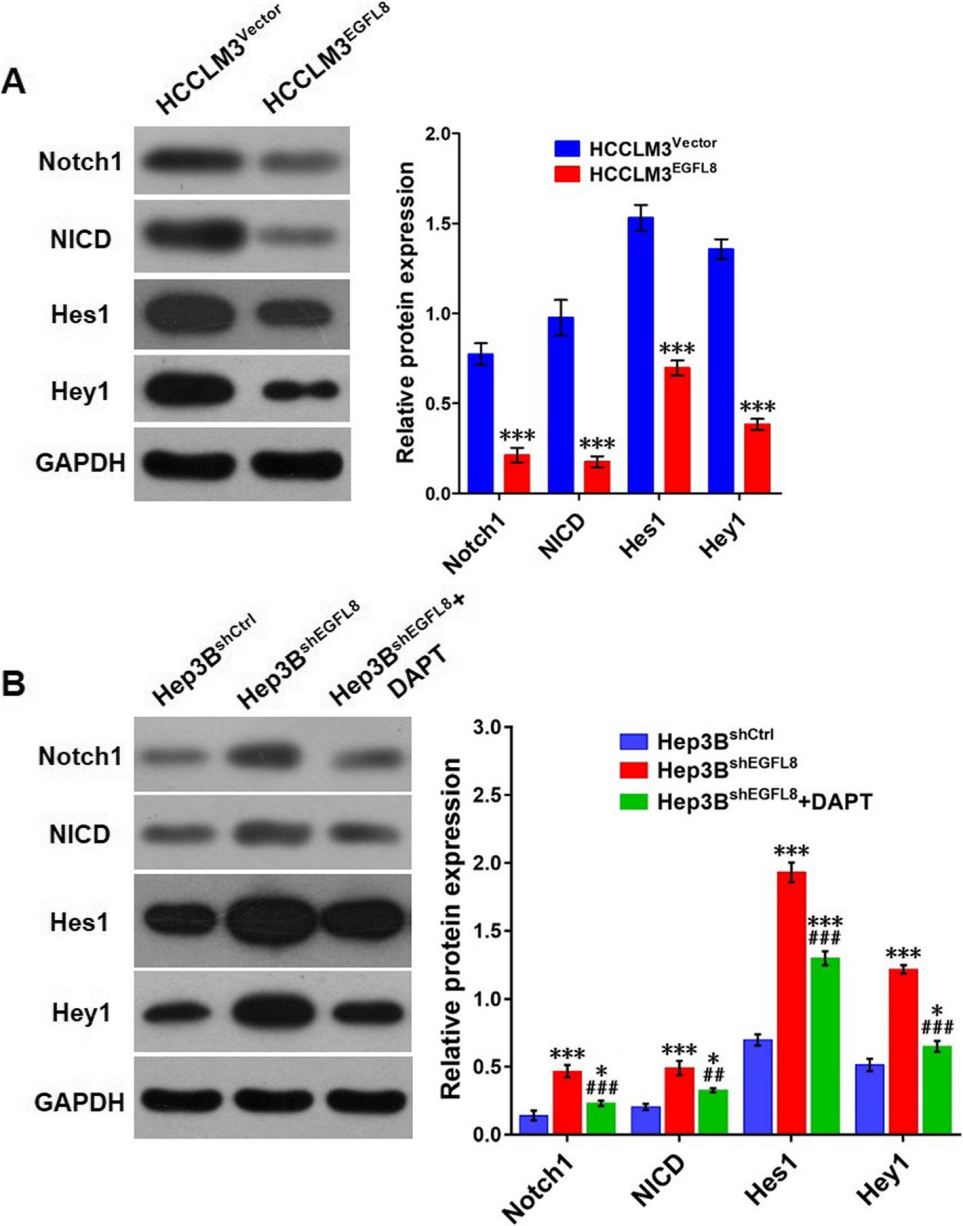
The expression levels of Notch signaling pathway in EGFL8 overexpressed or depleted HCC cells. **a** Western blot results showed the expression of Notch1, NICD, Hes1, and Hey1 in HCCLM3^EGFL8^ cells was obviously lower than that in HCCLM3^Vecter^ cells. All the data were showed as mean ± SEM. ***, *P* < 0.001, versus HCCLM3^Vector^ cells. Full-length blots are presented in Supplementary Fig. 2. **b** Western blot results showed the expression of Notch1, NICD, Hes1, and Hey1 in Hep3B^shEGFL8^ cells was markedly higher than that in Hep3B^shCtrl^ cells. However, the expression levels of Notch1 signaling pathway in Hep3B^shEGFL8^ cells were significantly decreased after DAPT treatment. All the data were showed as mean ± SEM. *, *P* < 0.05, versus Hep3B^shCtrl^ cells; ***, *P* < 0.001, versus Hep3B^shCtrl^ cells; ^##^, *P* < 0.01, versus Hep3B^shEGFL8^; ^###^, *P* < 0.001, versus Hep3B^shEGFL8^. Full-length blots are presented in Supplementary Fig. 2

For a recent study of Subhan et al. suggested the involvement of the Notch signaling pathway in the role of EGFL8 [22], we explored the expression of the Notch signaling pathway in EGFL8 overexpressed or depleted liver cancer cells. Our results showed that the expression levels of Notch1, NICD, Hes1, and Hey1 were obviously suppressed in EGFL8 overexpressed HCCLM3 cells (Fig. 4a) but significantly elevated in EGFL8 depleted Hep3B cells (Fig. 4b), suggested EGFL8 as a suppressing modulator for the Notch signaling pathway. When we treated EGFL8 depleted Hep3B cells with DAPT, a γ-secretase inhibitor, the up-regulated expression of the Notch signaling pathway induced by EGFL8 depletion was remarkably declined (Fig. 4b) and the results from wound healing assay showed the migration of Hep3B cells was obviously weakened (for 24 h: 9.00% ± 1.16% versus 25.67% ± 1.33%, *P <* 0.001; for 72 h: 16.00% ± 0.58% versus 38.67% ± 2.03%, *P <* 0.001; Fig. 2c). Moreover, the Transwell assay also evidenced a reduced number of Hep3B^shEGFL8^ cells invaded pass through the matrigel after DAPT treatment (69.33 ± 3.18 versus 105.30 ± 6.98, *P* = 0.0057; Fig. 2d). MTT assay showed no influence of DAPT treatment on the proliferation of Hep3B^shEGFL8^ cells (Fig. 2e), while the apoptosis of DAPT-treated Hep3B^shEGFL8^ cells was mildly higher than those without DAPT treatment (4.10% ± 0.29% versus 2.77% ± 0.07%, *P* = 0.0054; Fig. 2f).

## Discussion

Although the expression of EGFL8 is known to be highly expressed in kidney, brain, thymus, and lung of adult mouse [13], its expression pattern in human HCC tissue remains unknown. The present study therefore firstly showed the downregulated expression of EGFL8 in HCC tissues and this down-regulation was evidenced in most (73.81%) of the HCC patients. These results are in accordance with our previous results, showing the decreased EGFL8 expression in colorectal and gastric cancer tissues, and further indicate the down-regulation of EGFL8 as a common pathological event in the development of malignancies of human digestive system [14, 15]. Recently, Lu et al. [23] has showed a consistently high methylation level of EGFL8 in 18 cell lines from 9 types of human tumors such as colorectal cancer, suggesting hypermethylation as a potential cause of the down-regulation of EGFL8 in human cancers.

When correlating the down-regulation of EGFL8 with the clinicopathologic features of HCC, we found that EGFL8 was more often down-regulated in HCC with multiple nodes than those with solitary node. In addition, EGFL8 down-regulation was also closely associated with vein invasion. For these two clinicopathological characteristics are all acknowledged markers for metastasis of HCC [24, 25], it is unsurprisingly that EGFL8 down-regulation was also closely related to high TNM stage, which is also in accord with our previous studies indicating the correlation between EGFL8 down-regulation and high TNM stage of colorectal and gastric cancers. Together, these results therefore suggested that EGFL8 might be involved in the metastasis of HCC and the expression of EGFL8 may be down-regulated in accompany with the progression of human digestive cancers including HCC. For TNM stage is a well-accepted marker for the prognosis of HCC [2, 6], our Kaplan- Meier analysis also found that the HCC patients with low EGFL8 expression had worse overall survival and progression free survival than those with high EGFL8 expression, suggesting EGFL8 as a potential prognostic biomarker for HCC, which, of cause, should be further verified in the future studies.

We also determined the expression pattern of EGFL8 in a normal liver cell line and three liver cancer cell lines with different metastatic abilities by RT-qPCR. The expression of EGFL8 were all down-regulated in all three liver cancer cell lines compared with the normal liver cell line, which confirmed the down-regulation of EGFL8 in HCC tissues. Of particular interest is the relationship between EGFL8 down-regulation and the metastatic capacity of these liver cancer cell lines. Hep3B and HepG2 cells exhibit almost non- and low- metastatic ability respectively [26, 27], whereas HCCLM3 cells has a high metastatic ability to form lung metastases by either subcutaneous or orthotopic inoculation [28]. EGFL8 expression declined in these three liver cancer cell lines in the order by their metastatic potential descending, suggesting an obviously involvement of EGFL8 in the metastasis of HCC.

To understand the role of EGFL8 in HCC development, we employed lentivirus-mediated gene transfer or shRNA to enhance or suppress the EGFL8 expression in HCCLM3 or Hep3B cell line, which had the lowest or highest expression level of EGFL8 among the three liver cancer cell lines we tested respectively. Our results of these gain- and loss-of-function experiments showed at the first time that EGFL8 obviously suppressed the metastatic capacity of HCC cells. The negative regulation of EGFL8 in metastasis of liver cancer cells was further validated by an HCC metastasis mouse model, which showed that the pulmonary metastatic ratio in the EGFL8 overexpression group was significantly lower than the controlled group, indicating EGFL8 as an important modulator in the metastasis of HCC. In view of the possibility that cell proliferation could influence cell migration, we carried out an MTT assay to compare the proliferation of EGFL8 overexpressed HCCLM3 cells or EGFL8 depleted Hep3B cells with the corresponding negative control cells. Interestingly, there was no significant difference was found between the growth curves of these two groups of liver cancer cells. However, our results indeed showed a weakly promotive effect of EGFL8 on the apoptosis of liver cancer cells. These data are consistent with a recent study which showed mouse recombinant EGFL8 protein could inhibit the survival of mouse thymocytes [22].

For Subhan et al. [22] have found that EGFL8 restrains mouse thymocyte proliferation and induces apoptosis by negative regulating the expression of the Notch downstream proteins including Hes1 and Hey1, which are also involved in the metastasis of HCC [21, 27]. We therefore presumed that EGFL8 might regulate the metastasis and apoptosis of liver cancer cells through inhibiting the Notch signaling pathway. To verify this hypothesis, we detected the expression levels of Notch signaling pathway including Notch1, NICD, Hes1, and Hey1 in liver cancer cells by Western blot, and the results exhibited a significant decrease of these proteins in EGFL8 overexpressed HCCLM3 cells and an obvious increase of their expression in EGFL8 depleted Hep3B cells. Moreover, IHC assay also evidenced a markedly down- regulation of Notch1 protein in the xenograft tumors came from EGFL8 overexpressed HCCLM3 cells. All these in vitro as well as in vivo data have indicated an inhibition effect of EGFL8 on Notch signaling pathway in HCC. Furthermore, we treated EGFL8 depleted Hep3B cells with DAPT, an inhibitor of Notch signaling pathway [21, 29], and found this treatment obviously inhibit the expression of Notch signaling pathway and significantly suppressed the migration, invasion, and survival of Hep3B cells which had been remarkably enhanced by EGFL8 knockdown, suggesting the modulation of EGFL8 on liver cancer cell metastasis and apoptosis is, at least partially, depend on the inhibition of Notch signaling pathway. However, the specific molecular mechanism underlying the regulation of EGFL8 on Notch signaling pathway remains to be further explored.

## Conclusion

The present study has provided the first evidence to show the down-regulation of EGFL8 in HCC tissues correlates significantly to the development and prognosis of this malignancy. Furthermore, we have demonstrated the suppressing effects of EGFL8 on HCC cell metastasis, invasion and survival through regulating the Notch signaling pathway, indicating EGFL8 as a novel therapeutic target and a potential prognostic marker for HCC.

## Supplementary Information

**Additional file 1.**

**Additional file 2.**

## Data Availability

All data generated or analysed during this study are included in this published article.

## Abbreviations

AFP: Α-fetoprotein
ANLT: Adjacent non-tumorous liver tissue
ANOVA: Analysis of variance
CT: Computed tomography
DAPT: N-[N-(3,5-Difluorophenacetyl-L- alanyl)]-(S)-phenylglycine t-butyl ester
DAB: 3,3-diaminobenzidine tetrahydro- chloride
DMEM: Dulbecco’s modified Eagle’s medium
DMSO: Dimethylsulfoxide
EGFL8: Epidermal growth factor-like domain 8
FBS: Fetal bovine serum
GAPDH: Glyceraldehyde-3-phosphate dehydrogenase
HCC: Hepatocellular carcinoma
Hes1: Hairy and enhancer of split homolog-1
Hey1: Hairy and Enhancer-of-split related with YRPW motif 1
IHC: Immunohisto- chemistry
MTT: 3-(4,5- Dimethylthiazol-2-yl)-2,5- diphenyltetrazolium bromide
NICD: Notch intracellular domain
PDS: Phosphate buffer saline
PVDF: Polyvinylidene Fluoride
RT-qPCR: Reverse transcription-quantitative polymerase chain reaction
SEM: Standard error of mean
shRNA: Short hairpin RNA

## References

[1] Bray F, Ferlay J, Soerjomataram I, Siegel RL, Torre LA, Jemal A (2018). Global cancer statistics 2018: GLOBOCAN estimates of incidence and mortality worldwide for 36 cancers in 185 countries. CA Cancer J Clin.

[2] Qiu J, Peng B, Tang Y, Qian Y, Guo P, Li M, Luo J, Chen B, Tang H, Lu C, Cai M, Ke Z, He W, Zheng Y, Xie D, Li B, Yuan Y (2017). CpG methylation signature predicts recurrence in early-stage hepatocellular carcinoma: results from a multicenter study. J Clin Oncol.

[3] Zhang Y, Li D, Feng F, An L, Hui F, Dang D, Zhao Q (2017). Progressive and prognosis value of notch receptors and ligands in hepatocellular carcinoma: a systematic review and meta-analysis. Sci Rep.

[4] Wang FS, Fan JG, Zhang Z, Gao B, Wang HY (2014). The global burden of liver disease: the major impact of China. Hepatology..

[5] Giordano S, Columbano A (2014). Met as a therapeutic target in HCC: facts and hopes. J Hepatol.

[6] Llovet JM, Ricci S, Mazzaferro V, Hilgard P, Gane E, Blanc JF, de Oliveira AC, Santoro A, Raoul JL, Forner A, Schwartz M, Porta C, Zeuzem S, Bolondi L, Greten TF, Galle PR, Seitz JF, Borbath I, Häussinger D, Giannaris T, Shan M, Moscovici M, Voliotis D, Bruix J (2008). Sorafenib in advanced hepatocellular carcinoma. N Engl J Med.

[7] Hu CT, Wu JR, Cheng CC, Wu WS (2017). The therapeutic targeting of HGF/c-met signaling in hepatocellular carcinoma: alternative approaches. Cancers (Basel).

[8] Vilchez V, Turcios L, Marti F, Gedaly R (2016). Targeting Wnt/β-catenin pathway in hepatocellular carcinoma treatment. World J Gastroenterol.

[9] Peng YF, Shi YH, Ding ZB, Ke AW, Gu CY, Hui B, Zhou J, Qiu SJ, Dai Z, Fan J (2013). Autophagy inhibition suppresses pulmonary metastasis of HCC in mice via impairing anoikis resistance and colonization of HCC cells. Autophagy..

[10] Wahid B, Ali A, Rafique S, Idrees M (2017). New insights into the epigenetics of hepatocellular carcinoma. Biomed Res Int.

[11] Parker LH, Schmidt M, Jin SW, Gray AM, Beis D, Pham T, Frantz G, Palmieri S, Hillan K, Stainier DYR, de Sauvage FJ, Ye W (2004). The endothelial-cell-derived secreted factor Egfl7 regulates vascular tube formation. Nature..

[12] Wu F, Yang LY, Li YF, Ou DP, Chen DP, Fan C (2009). Novel role for epidermal growth factor-like domain 7 in metastasis of human hepatocellular carcinoma. Hepatology..

[13] Fitch MJ, Campagnolo L, Kuhnert F, Stuhlmann H (2004). Egfl7, a novel epidermal growth factor-domain gene expressed in endothelial cells. Dev Dyn.

[14] Wu F, Shirahata A, Sakuraba K, Kitamura Y, Goto T, Saito M, Ishibashi K, Kigawa G, Nemoto H, Sanada Y, Hibi K (2011). Down-regulation of EGFL8: a novel prognostic biomarker for patients with colorectal cancer. Anticancer Res.

[15] Wu F, Shirahata A, Sakuraba K, Kitamura Y, Goto T, Saito M, Ishibashi K, Kigawa G, Nemoto H, Sanada Y, Hibi K (2011). Down-regulation of EGFL8: a novel biomarker for advanced gastric cancer. Anticancer Res.

[16] Ge XC, Wu F, Li WT, Zhu XJ, Liu JW, Wang BL (2017). Upregulation of WEE1 is a potential prognostic biomarker for patients with colorectal cancer. Oncol Lett.

[17] Wu F, Li TY, Su SC, Yu JS, Zhang HL, Tan GQ, Liu JW, Wang BL (2017). STC2 as a novel mediator for Mus81-dependent proliferation and survival in hepatocellular carcinoma. Cancer Lett.

[18] Wu F, Su SC, Tan GQ, Yan L, Li TY, Zhang HL (2016). Mus81 knockdown improves chemosensitivity of hepatocellular carcinoma cells by inducing S-phase arrest and promoting apoptosis through CHK1 pathway. Cancer Med.

[19] Wang W, Wu F, Fang F, Tao YM, Yang LY (2008). RhoC is essential for angiogenesis induced by hepatocellular carcinoma cells via regulation of endothelial cell organization. Cancer Sci.

[20] Wu F, Su SC, Tan GQ, Yan L, Li TY, Zhang HL, Yu JS, Wang BL (2017). Mus81 knockdown sensitizes colon cancer cells to chemotherapeutic drugs by activating CHK1 pathway. Clin Res Hepatol Gastroenterol.

[21] You K, Sun P, Yue Z, Li J, Xiong W, Wang J (2017). NOR1 promotes hepatocellular carcinoma cell proliferation and migration through modulating the notch signaling pathway. Exp Cell Res.

[22] Subhan F, Yoon TD, Choi HJ, Muhammad I, Lee J, Hong C (2013). Epidermal growth factor-like domain 8 inhibits the survival and proliferation of mouse thymocytes. Int J Mol Med.

[23] Lu TP, Chen KT, Tsai MH, Kuo KT, Hsiao CK, Lai LC (2014). Identification of genes with consistent methylation levels across different human tissues. Sci Rep.

[24] Choi JH, Chung WJ, Bae SH, Song DS, Song MJ, Kim YS, Yim HJ, Jung YK, Suh SJ, Park JY, Kim DY, Kim SU, Cho SB (2018). Randomized, prospective, comparative study on the effects and safety of sorafenib vs. hepatic arterial infusion chemotherapy in patients with advanced hepatocellular carcinoma with portal vein tumor thrombosis. Cancer Chemother Pharmacol.

[25] Okamura Y, Sugiura T, Ito T, Yamamoto Y, Ashida R, Aramaki T, Uesaka K (2018). The predictors of microscopic vessel invasion differ between primary hepatocellular carcinoma and hepatocellular carcinoma with a treatment history. World J Surg.

[26] Wang XQ, Zhang W, Lui EL, Zhu Y, Lu P, Yu X (2012). Notch1- Snail1-E-cadherin pathway in metastatic hepatocellular carcinoma. Int J Cancer.

[27] Ma Y, Bian J, Zhang F (2016). Inhibition of perillyl alcohol on cell invasion and migration depends on the notch signaling pathway in hepatoma cells. Mol Cell Biochem.

[28] Li Y, Tang Y, Ye L, Liu B, Liu K, Chen J (2003). Establishment of a hepatocellular carcinoma cell line with unique metastatic characteristics through in vivo selection and screening for metastasis-related genes through cDNA microarray. J Cancer Res Clin Oncol.

[29] Liao B, Zhou H, Liang H, Li C (2017). Regulation of ERK and AKT pathways by hepatitis B virus X protein via the Notch1 pathway in hepatocellular carcinoma. Int J Oncol.

