# Supplementary material for: Down-regulation of EGFL8 regulates migration, invasion and apoptosis of hepatocellular carcinoma through activating Notch signaling pathway
Source: BMC Cancer. 2021 Jun 15;21:704. doi: 10.1186/s12885-021-08327-0 (PMC8207656; doi:10.1186/s12885-021-08327-0)

Original gels and blots of Notch1, NICD, Hes1, Hey1 and GAPDH in HCCLM3 cells and Hep3B cells (Corresponding to Fig. 4A&B in the manuscript).


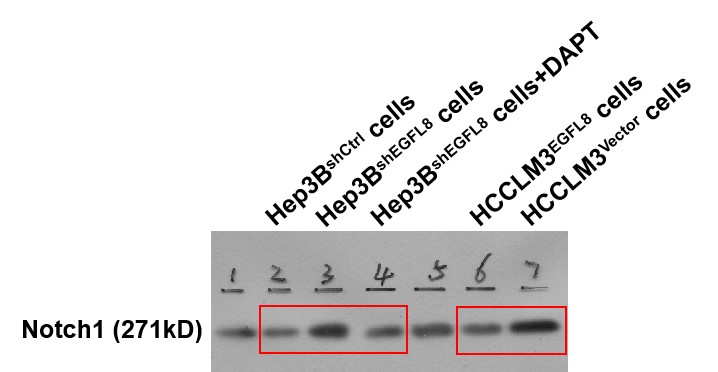


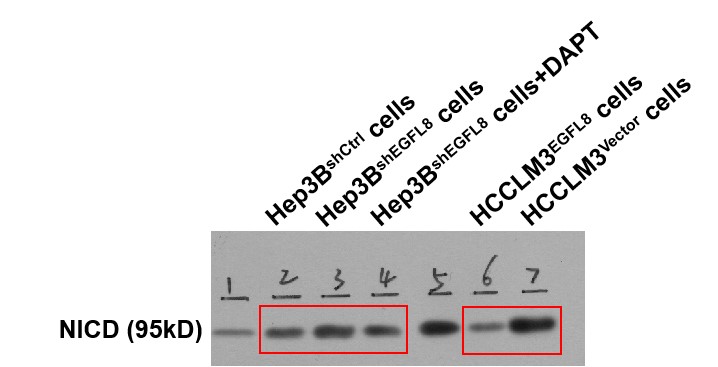


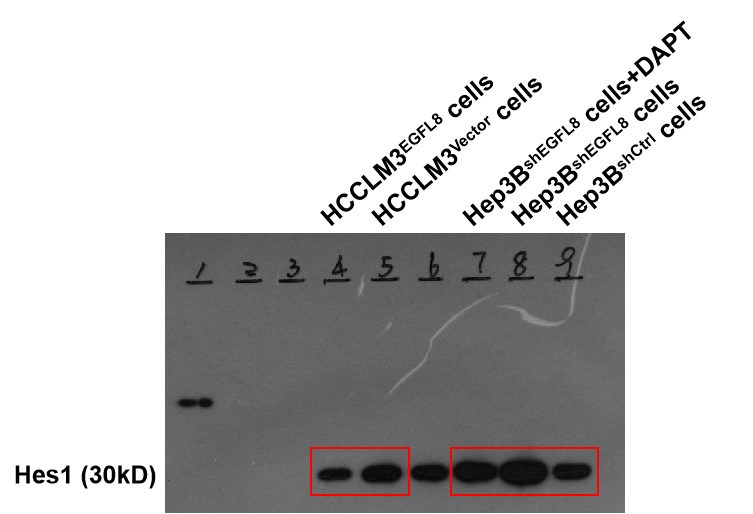


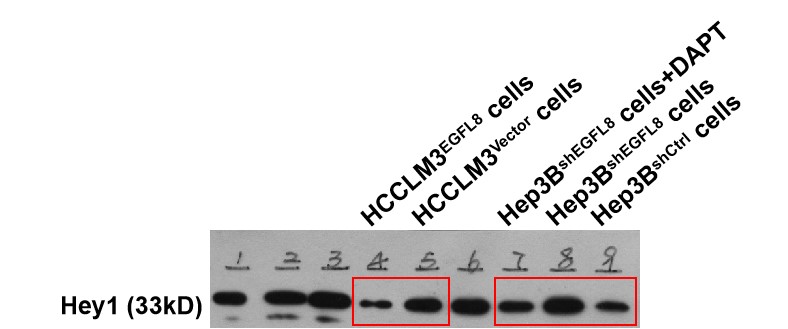


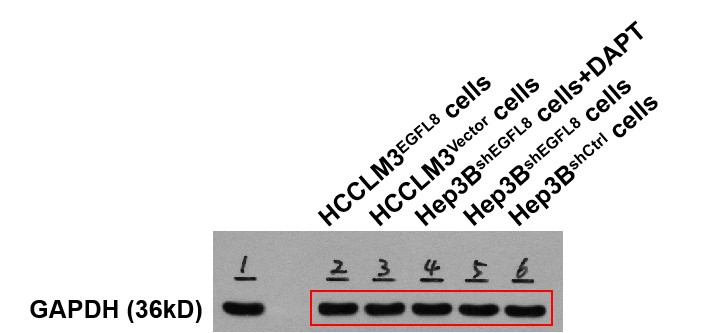

Supplement: Supplementary file 2 — Additional file 2. [file 12885_2021_8327_MOESM2_ESM.docx]
